# Western Hunter-Gatherer genetic ancestry contributes to human longevity in the Italian population

**DOI:** 10.1007/s11357-025-02043-4

**Published:** 2025-12-13

**Authors:** Stefania Sarno, Vincenzo Iannuzzi, Marco Sazzini, Chiara Pirazzini, Maria Giulia Bacalini, Davide Gentilini, Giuseppe Passarino, Daniela Mari, Daniela Monti, Benedetta Nacmias, Sandro Sorbi, Davide Pettener, Donata Luiselli, Claudio Franceschi, Paolo Garagnani, Cristina Giuliani

**Affiliations:** 1 Laboratory of Molecular Anthropology & Centre for Genome Biology, Department of Biological, Geological and Environmental Sciences, University of Bologna, Via Selmi 3, 40126 Bologna, Italy; 2 Alma Mater Research Institute On Global Challenges and Climate Change (Alma Climate), University of Bologna, Bologna, Italy; 3 Department of Medical and Surgical Sciences (DIMEC), University of Bologna, Bologna, Italy; 4 Department of Biomedical and Neuromotor Sciences (DIBINEM), University of Bologna, Bologna, Italy; 5 Department of Brain and Behavioral Sciences, University of Pavia, 27100 Pavia, Italy; 6 Bioinformatics and Statistical Genomics Unit, Istituto Auxologico Italiano IRCCS, Milan, Cusano Milanino Italy; 7 Department of Biology, Ecology and Earth Sciences, University of Calabria, Rende, Italy; 8 Fondazione Ca’ Granda, IRCCS Ospedale Maggiore Policlinico, Milan, Italy; 9 Department of Experimental and Clinical Biomedical Sciences “Mario Serio”, University of Florence, Florence, Italy; 10 Department of Neuroscience, Psychology, Drug Research and Child Health, University of Florence, Florence, Italy; 11 Department of Cultural Heritage (DBC), University of Bologna, Ravenna Campus, Ravenna, Italy; 12 Institute of Biogerontology and Research Center in Artificial Intelligence, Lobachevsky University, Nizhny Novgorod, Russia

**Keywords:** Italian centenarians, Western Hunter-Gatherers, Longevity, Paleogenomics, Human genetics, Biodemography

## Abstract

The genetics of human longevity has been primarily studied using classical methods developed in genome-wide association studies. With the recent advances in paleogenomics, it is now possible to investigate to what extent ancient population ancestries contribute to complex traits. In this study, we explored the role of ancient genetic components in human longevity by focusing on the Italian Peninsula, whose genetic heritage includes several past genetic ancestries that have contributed to the current European genetic make-up. We analyzed genome-wide data of 333 Italian centenarians and 690 geographically matched healthy controls, and compared their genetic composition to 103 ancient genomes representative of the main past European population ancestries. Our findings indicate that Italian centenarians have a higher genetic affinity with Western Hunter-Gatherer (WHG)-related ancestry compared to controls, according to PCA and f4-statistics. Logistic regression models based on supervised admixture revealed a significant association between higher WHG ancestry and the centenarian status. Additionally, residual-based predictive analysis showed that centenarians exhibit a significantly higher WHG contribution independent of the genetic structuring of the general Italian population. By painting the chromosomes of modern Italians, we also showed a significantly higher number of WHG alleles at pro-longevity SNPs. In the present study, we demonstrate the contribution of ancient genetic components to the longevity phenotype. In particular, we showed a greater contribution from Western Hunter-Gatherer-related ancestry to Italian centenarians, thus suggesting that this pre-Neolithic genetic component, which has been linked to population shifts occurring within Europe after the Last Glacial Maximum, could be beneficial for longevity today.

**Graphical Abstract:**

**Figure:**
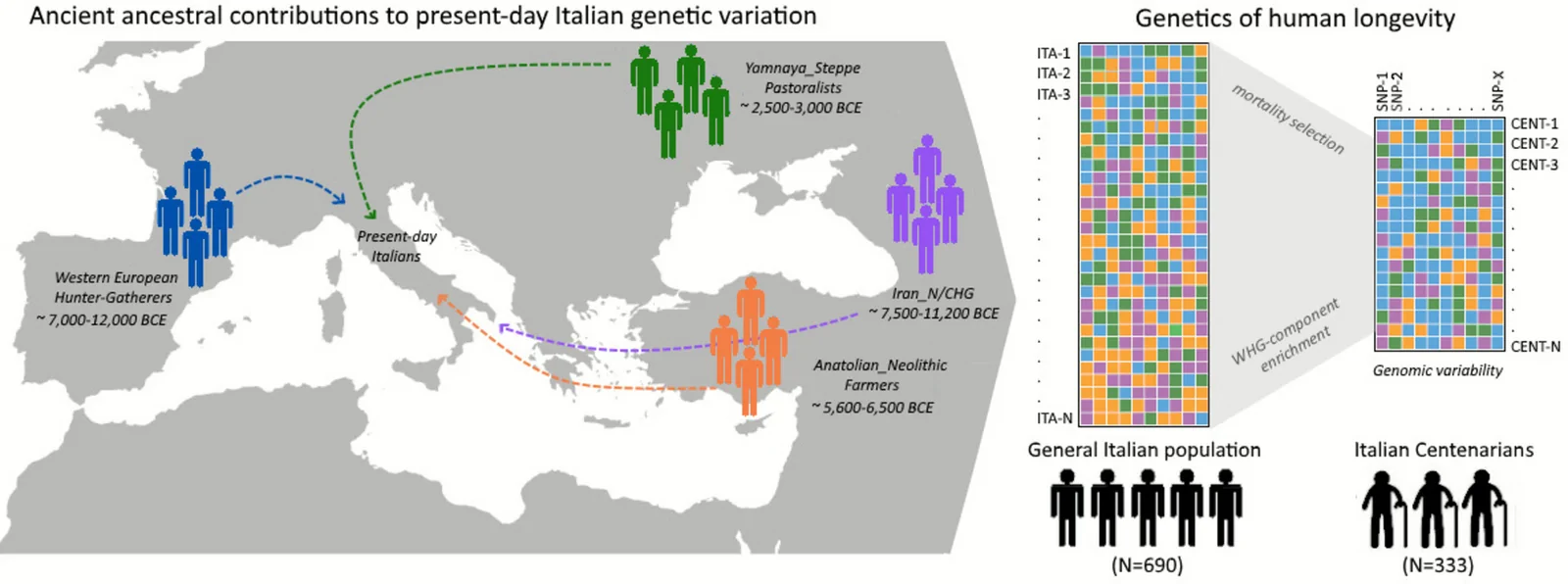

**Supplementary Information:**

The online version contains supplementary material available at https://doi.org/10.1007/s11357-025-02043-4.

## Introduction

Human longevity is a complex trait influenced by multiple genetic and environmental factors [1, 2]. Over the past decades, various methodological approaches have been employed to investigate its genetic basis. Among them, large-scale genome-wide association studies (GWAS) including multiple human populations, have played a central role in identifying genetic variants associated with increased longevity susceptibility [3–6]. However, recent research has emphasized that the genetics of human longevity also exhibit population-specific characteristics, suggesting that distinct demographic and evolutionary dynamics may have contributed to influence the genetic architecture of this complex trait in different human groups [1, 2, 7]. In this context, emerging approaches grounded in population genetics and paleogenomics can offer a powerful framework for investigating how past demographic processes may have contributed to shape the genetics of exceptional longevity in contemporary humans.

Recent advances in paleogenomics have substantially increased the number of studies leveraging ancient DNA (aDNA) to estimate the contribution of distinct ancestral components to modern phenotypes and complex traits, thereby elucidating how ancient demographic and evolutionary processes continue to influence present-day patterns of human health and disease susceptibility [8–11]. However, such an approach in human longevity studies is still limited. In fact, while some studies have applied evolutionary approaches and explored the relationship between ancient ancestry and longevity, they have primarily focused on a limited set of candidate genes critical for human longevity, such as FOXO3 or APOE [12–14]. At the same time, previous genome-wide studies that estimated the influence of ancient ancestries on present-day phenotypes [8, 11, 15] did not consider the most appropriate phenotype model for longevity, which focuses on centenarians or, more generally, on individuals corresponding to the 99th survival percentile of the birth cohort within the analysed population [3, 16]. Consequently, the broad genome-wide contribution of ancestral components to extreme longevity remains to be comprehensively investigated.

To fill this gap, the present study takes advantage of the growing availability of ancient genome-wide data to investigate whether, and to what extent, ancient ancestral populations may have contributed to the genetic basis of human longevity. To achieve this goal, we specifically focused on the Italian population, which may serve as an ideal case study as it minimizes potential confounding effects related to different population histories, while at the same time, its present-day genetic makeup recapitulates broader patterns of genetic variation observed across Europe [17–23]. In fact, previous investigations into the genetic history of Italy have largely suggested that the modern Italian gene pool encapsulates the contribution of at least four major ancestral genetic components that formed the current European genetic landscape, namely: (1) the European Mesolithic hunter-gatherers; (2) the early Neolithic farmers spreading from Anatolia; (3) the Bronze-Age herders from the Pontic-Caspian Steppe associated with the Yamnaya culture [24, 25]; and (4) a further non-Steppe genetic component related to Caucasian hunter-gatherers and Iranian Neolithic-related ancestry, carried along the Mediterranean shores at the same time but independently from the Bronze-Age Steppe migrations [22, 23, 26].

Within this framework, in the present study, we analyzed genome-wide data of 333 Italian centenarians (mean age: 100.4 ± 1.4 years) and 690 geographically matched healthy controls (mean age: 49.81 ± 12.76 years) from across the Italian Peninsula, and we directly compared them with a comprehensive panel of ancient genomes representative of the four above-mentioned ancient genetic ancestries to specifically explore possible ancestral contributions to the human longevity phenotype and to better understand how past demographic processes may still echo in the genetic makeup of present-day long-lived individuals.

## Materials and methods

### Sample description and data processing

In this study, we analyzed genome-wide data of 333 centenarians (*CENT*; mean age: 100.4 ± 1.4 years; 258 female, 75 male) and 690 geographically matched controls (*ITA*; mean age: 49.81 ± 12.76 years; 336 female, 352 male) from Italy, excluding Sardinia. The genotyping data for both cohorts were previously generated in our study [7]. Centenarians, all born between 1901 and 1913, were selected as individuals who survived beyond the 99th percentile of their birth cohort, and they were sampled at three different Italian recruitment centers in Northern Italy (*N* = 66), Central Italy (*N* = 176), and Southern Italy (*N* = 91). The control subjects were selected to be representative of the Italian population following strict biodemographic criteria to include only individuals at least three generations native to a given district with all grandparents originating from the same province [21], and matching the geographic distribution of centenarians among Northern (*N* = 131), Central (*N* = 403), and Southern (*N* = 156) Italy [7].

Genomic DNA of Italian individuals was extracted from blood samples using the QIAamp 96 DNA Blood Kit. DNA quantification was performed using the Quant-iT dsDNA Broad-Range Assay Kit (Invitrogen Life Technologies, Carlsbad, CA, USA) or by the Quant-iT PicoGreen dsDNA Assay Kit (Thermo Fisher Scientific) according to the manufacturer’s protocols. Then, 200 ng of DNA was genotyped for 542,585 genetic markers with the Illumina CoreExomeChip v.1.1 array (San Diego, CA, USA). Quality control checks (QCs) on this modern “Italian dataset” were performed according to the pipelines described previously [7].

Subsequently, to explore possible ancestral genetic contributions to human longevity, the Italian *CENT* and *ITA* sample sets were combined with a representative panel of ancient individuals (Supplementary File 1: Supplementary Table 1) selected from those cited by the Allen Ancient DNA Resource [27] (AADR v44.3—release data: Jan 20, 2021) to account for the main basal ancient European ancestries. In particular, we included ancient samples known to represent homogeneous ancestral groups and excluded from the analyses any individual annotated as low-coverage, contaminated, outliers, or genetically related. In detail, we specifically considered 26 Neolithic Farmers from Anatolia (*Turkey_N*), 16 Yamnaya Pastoralists from the Pontic Steppes (*Yamnaya_EBA*), 2 Caucasian hunter-gatherers, and 12 Iran Neolithic *(Iran_N*), as well as 47 individuals representative of the Western European Hunter-Gatherer (*WHG*) ancestry, namely 10 Romania Iron-Gates, 33 Serbia Iron-Gates, and 4 Italian Mesolithic samples (Supplementary File 1: Supplementary Table 1). For all paleogenomes, diploid genotypes were called from BAM files using the *snpAD* software [28], by considering a minimum base quality (Phred-scaled) of 30 and a minimum mapping quality (Phred-scaled) of 30.

The merging between the modern Italian dataset and the 103 ancient selected individuals resulted in a merged genotyping dataset encompassing 1126 samples and 498,285 common autosomal SNPs, after excluding sexual chromosomes. This dataset, unless otherwise specified, was used for all subsequent analyses aimed at comparing *CENT* and *ITA* groups in relation to the above-mentioned ancient genetic ancestries. Each analysis was performed by contrasting centenarians with the large Italian control population, while focusing on each ancestral component at a time. This strategy ensured that the identification of centenarian-specific genetic signals was independent of ancestral group size.

Furthermore, for the genotype-based analyses requiring LD-pruning, we excluded one SNP for each pair of loci showing *r*^2^ values higher than 0.4 within a 200–SNPs window, sliding 25 loci at a time (PLINK option *–indep-pairwise 200 25 0.4*), for a total of 105,337 SNPs retained after pruning.

To assess the robustness of the results and control for sex-related differences in sample composition, all the analyses described below were also conducted separately on the female subgroup (centenarians: *n* = 258 vs. controls: *n* = 336). Male-specific analyses, however, were not performed due to the limited number of centenarian men (*n* = 75).

### PCA projection analysis

A Principal Component Analysis (PCA) was initially performed on the joint dataset of ancient and modern individuals (Supplementary File 1: Supplementary Table 1 and Supplementary Table 2) using the *smartpca* function of the EIGENSOFT package [29] with the “*lsqproject: YES*” option. In particular, we computed principal components (PCs) on a modern dataset including our Italian samples together with 1,257 previously published present-day individuals from diverse West Eurasian populations extracted from the Human Origin (HO) panel, and projected the selected ancient individuals onto the first two PCs of the obtained Euro–Mediterranean genetic landscape.

After performing PCA, the Euclidean distances between the individuals from the *CENT* or *ITA* group and the centroids of the four ancestral populations (*WHG*, *Iran_N*, *Turkey_N*, and *Yamnaya_EBA*) were calculated in the above-mentioned two-dimensional space defined by the first two principal components (PC1 and PC2). Subsequently, the obtained distances of both *CENT* and *ITA* individuals to the centroids of each ancestral group were compared using the Wilcoxon rank-sum test to assess the presence of significant differences between the *CENT* and *ITA* cohorts.

### f4-statistics analysis

The genetic relationships between *CENT* and *ITA* with respect to each of the considered ancient population groups were assessed also by using f-statistics in the form of *D (CENT, ITA; Ancient, Mbuti).* The f4-statistics were computed using the *qpDstat* function (with the option “*f4mode: YES*”) from the ADMIXTOOLS package [30]. In particular, to address the sample size disparity between the 333 *CENT* and 690 *ITA* individuals, we performed random subsampling of 345 *ITA* individuals while maintaining the genetic, geographic, and sex distribution within the subsampled group. This procedure was repeated across 1000 iterations, and at each iteration, the Z-scores associated with the D-statistic were calculated by finally obtaining a distribution of Z-scores for each of the considered ancient population groups (*WHG*, *Turkey_N*, *Iran_N*, *Yamnaya_EBA*). Comparisons were considered statistically significant if the expected value of Z-score distributions exceeded an absolute value of 3.

### Supervised admixture

Individual-level ancestry proportions for the 333 *CENT* and 690 *ITA* were estimated using the supervised admixture approach implemented in the ADMIXTURE software [31], by considering the four ancient groups, i.e., Western Hunter-Gatherers (*WHG*), Turkey Neolithic (*Turkey_N*), Iran Neolithic *(Iran_N*), and Yamnaya Early Bronze Age (*Yamnaya_EBA*), as ancestral donor populations. The Q-matrix generated from the supervised admixture, representing the relative contributions of the four ancestral groups to each recipient individual’s genome, was then used as input data for two different analyses: (i) a logistic regression analysis and (ii) a residual-based predictive analysis.

#### Logistic regression analysis

The first analysis used logistic regression models to directly investigate associations between ancestry proportions and centenarian status. Separate models were fitted for each Ancestral Component (AC) adjusting for PC1 to account for population genetic structuring, which corresponds to the principal component capturing the north-to-south gradient of genetic variation within the Italian Peninsula in the PCA performed comparing centenarians and controls to ancient samples within the Euro-Mediterranean genetic landscape. AC predictors were standardized by subtracting the mean and dividing by the standard deviation in order to make them directly comparable. This procedure ensured that obtained coefficients reflect the change in the log-odds of the outcome (*CENT*) for a 1-standard deviation change in the predictor, thus facilitating comparisons across ACs. For each AC the following logistic model was specifically fitted: CENT∼AC+PC1, where the dependent variable (*CENT*) was binary, coded as 1 for *CENT* and 0 for *ITA*.

#### Residual-based predictive analysis

To complement the logistic regression analysis and provide a different perspective, a residual-based predictive framework was also employed. This method exploits the correlation between ancestry components (ACs) and the genetic structure (here represented by the PC1) in the Italian general population, and then uses linear regression models of ACs on PC1 in order to capture the variability in ancestry proportions that cannot be explained by the population's genetic structure.

**Fig. 1:**
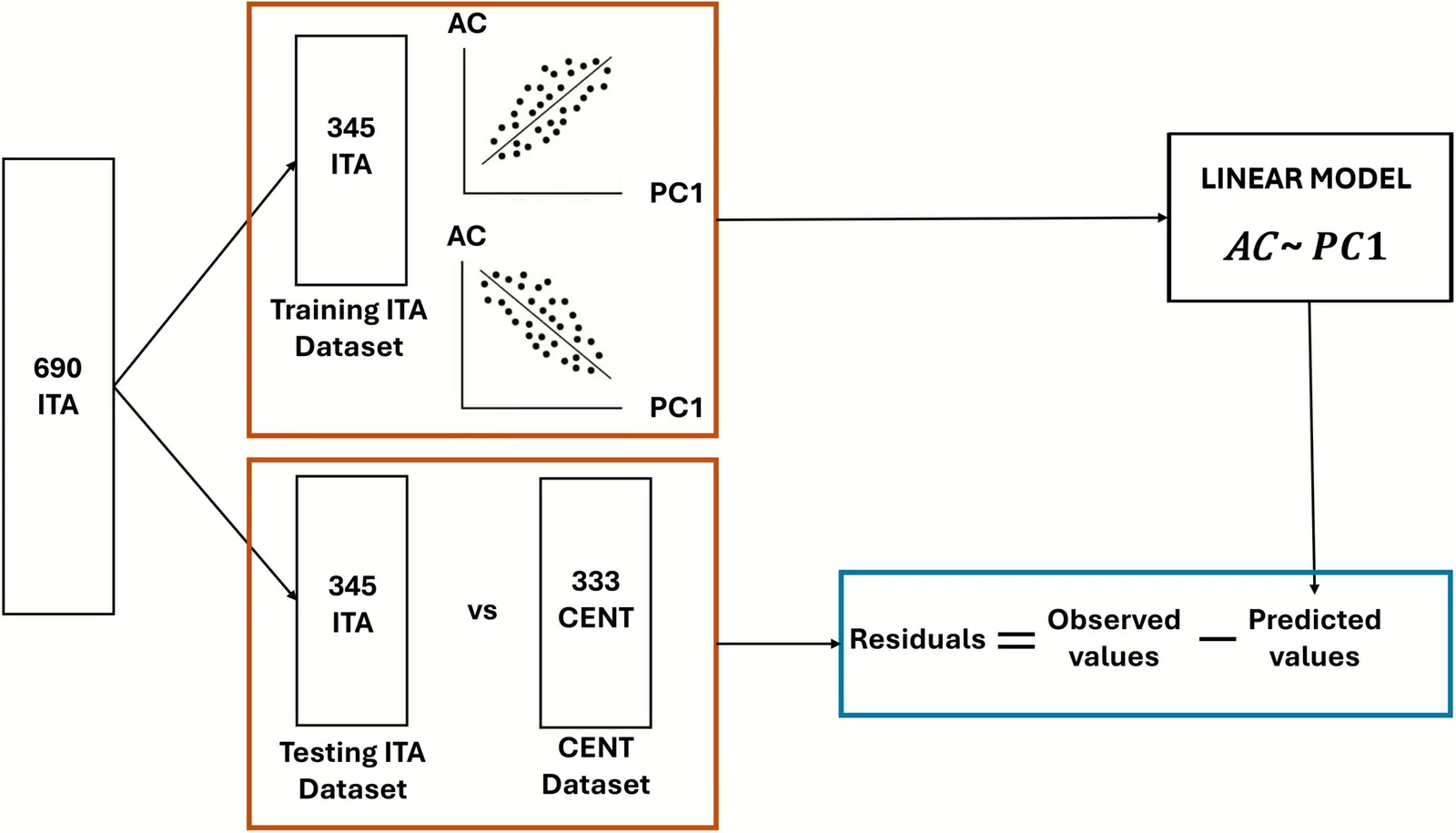
Analytical workflow for estimating deviations in ancestral component (AC) proportions after accounting for genetic structure. The *ITA* control group (*n* = 690) was randomly divided into a training set (*n* = 345) and a testing set (*n* = 345), while maintaining the genetic, geographic, and sex distribution of the original sample. In the training set, the correlations between AC proportions and the genetic structure (as measured by PC1) were assessed, and linear models were fitted to predict AC proportions as a function of PC1, separately for each AC. These models were then applied to the *CENT* and *ITA* testing sets to obtain predicted ancestry proportions. Residuals—defined as the difference between observed and predicted values—were finally computed and interpreted as deviations from expected AC proportions, after accounting for the genetic structure

To perform this analysis, the *ITA* controls were first randomly divided into a training (*n* = 345) and a testing (*n* = 345) dataset. In particular, to ensure the validity of the comparisons, the random subsampling of the original 690 *ITA* controls into two groups was performed by maintaining the matching in the genetic, geographic, and sex distribution between the training and testing datasets (Fig. 1). After assessing the correlation between ACs and the genetic structure (PC1) within the training *ITA* dataset, linear models were fitted in the training set to predict AC proportions as a function of PC1, separately for each AC. These models were then applied to both the *CENT* and the testing *ITA* sets to calculate predicted ancestry proportions. By fitting linear models in the training *ITA* set to predict the ancestry proportions based on PC1, we established baseline expectations for ancestry proportions in *CENT* and *ITA* separate testing sets. Residuals were then computed as the difference between observed and predicted ancestry proportions and were interpreted as deviations from expected AC proportions after accounting for the genetic structure captured by PC1 (Fig. 1). The residuals were then scaled to ensure that deviations in ancestry components could be interpreted on a standardized scale, allowing direct comparisons of the magnitude of residuals across ancestry components.

After assessing the normality assumption for the distribution of the residuals in both *CENT* and testing *ITA*, a *t*-test or a Wilcoxon test was finally performed to evaluate whether *CENT* exhibits systematic deviations in ancestry proportions that are not ascribable to the genetic structure and that distinguish them from the general Italian population. All analyses were implemented in the R software, by using the *caret* package for data partitioning, *glm* for logistic regression modelling, and *lm* for linear modelling.

### Chromopainter analysis

We used ChromoPainter v.2 (CP) [32] to infer ancient ancestry tracts in the genomes of present-day individuals. For this purpose, the “Italian dataset” composed of 333 *CENT* and 690 *ITA* was firstly merged with data derived from 69 individuals belonging to 35 modern European and Mediterranean populations, and 38 individuals belonging to modern Sub-Saharan African populations [33] in order to increase the phasing accuracy. This resulted in a new dataset including 305,218 SNPs and 1,130 modern human genomes. This dataset was subsequently integrated with the data of the 103 ancient individuals representing the main ancient European ancestries mentioned above, thus yielding a “combined dataset” overall comprising a total of 1,233 individuals and 296,923 SNPs.

The obtained combined dataset, encompassing both modern and ancient individuals, was phased with the SHAPEIT software v.2.17 [34, 35] by using default parameters and HapMap phase II b37 recombination maps [36]. Then, we exploited the haplotype-based approach implemented in CP to detect ancient ancestry in the genome of present-day *CENT* and *ITA*. CP indeed uses phased haplotype data to paint individual recipient chromosomes as a mosaic of genetic chunks inherited from a set of predefined donor individuals. Specifically, for each recipient haplotype, CP assigns the expected probability to copy from each donor population at each SNP. Therefore, we painted the 333 *CENT* and the 690 *ITA* individuals using the *WHG*, *Turkey_N*, *Yamnaya_EBA*, and *Iran_N* as donor populations, respectively. More precisely, we applied a two-step approach as described in [37]:

1. We initially conducted a first run of CP to estimate the prior copying probabilities (PCPs) for each recipient individual and each chromosome, by using 10 Expectation–Maximization (EM) steps. Given the different number of genotyped positions within and between the donor populations, we calculated a weighted average of PCPs across the genome, to estimate a unique genome-wide average PCP to be used in the next step of the analysis;
2. We then performed the main run of CP using the global mutation probability (*mu*), the recombination scaling constant (*N*_*e*_), and the genome-wide average PCP estimated from the first step.

For each recipient individual, a certain ancient-specific ancestry (i.e., *WHG*, *Anatolian_N*, *Yamnaya_EBA*, *Iran_N*) was assigned to SNPs with an expected probability to copy from the respective donor population greater than 0.85; “unknown ancestry” was instead assigned to SNPs with an expected probability to copy from donor populations lower than 0.85. After assessing the normality assumption in the distribution of the total number of SNPs of a certain ancestry, a *t*-test was performed to investigate eventual differences between the 333 *CENT* and 690 *ITA*.

#### Assessing ancestry-specific burden at pro-longevity SNPs

Based on ChromoPainter results, we then specifically focused on the copy probabilities associated with 18 pro-longevity SNPs (Supplementary File 1: Supplementary Table 3) that emerged from the association analysis between the 333 *CENT* and 690 *ITA* individuals, as reported in Giuliani et al. (2018). To prevent potential redundancy, linkage disequilibrium (LD) was evaluated among SNP pairs belonging to the same haploblock. In cases where two SNPs showed an *r*^2^ ≥ 0.7, one of them was removed in order to avoid biasing the results. Then, for each ancestral population (*WHG*, *Turkey_N*, *Yamnaya_EBA*, and *Iran_N*), we constructed a binary matrix to represent the “genetic composition” of each modern individual at each independent pro-longevity SNP with respect to the probability of copying from each donor population. More precisely, for each SNP of each individual’s haplotype, a value of “1” was assigned if the copy probability from a specific ancestral population exceeded 0.85, and a value of “0” was assigned if the probability was below this threshold. These binary matrices reflect the probability of each individual inheriting a particular pro-longevity allele from one of the considered ancestral populations. Subsequently, for each ancestral population, we created a new matrix by aggregating the binary data for each modern individual across their two haplotypes, thus indicating the total number of alleles “inherited” from each of the four ancestral populations. Specifically, a value of “0” was used to indicate no alleles copied from a given ancestral population, “1” for indicating one allele copied, and “2” for both alleles copied, at each pro-longevity SNP. These matrices were then used to perform a burden test for each ancestral component using the *SKAT* package [38, 39] in R. Specifically, after creating a null model for a dichotomous trait including PC1 as a covariate, we ran the *SKATBinary* function with *method* = “*Burden*” option and default settings. This test assessed whether the total burden, defined as the cumulative number of alleles inherited from each ancestral population at the pro-longevity SNPs, significantly differed between *CENT* and *ITA*.

## Results

We analysed genome-wide data of 333 Italian centenarians (*CENT*) and 690 geographically matched controls (*ITA*) in order to investigate whether their genetic make-up is different in terms of ancient genetic components tracing back to past population ancestries. To achieve this goal, we compared the genetic profiles of *CENT* and *ITA* with genomic data from a comprehensive panel of 103 ancient samples spanning the last 20,000 years (Supplementary File 1: Supplementary Table 1), selected for representing the main basal ancestries known to have left a significant impact on the European genetic pool, i.e., the Mesolithic Western European Hunter-Gatherers (*WHG*), the Anatolian Neolithic Farmers (*Turkey_N*), the Bronze-Age Steppe herders (*Yamnaya_EBA)*, and Caucasian hunter-gatherers and Iran Neolithic related groups (*Iran_N*).

### Genetic affinity to ancient human populations

**Fig. 2:**
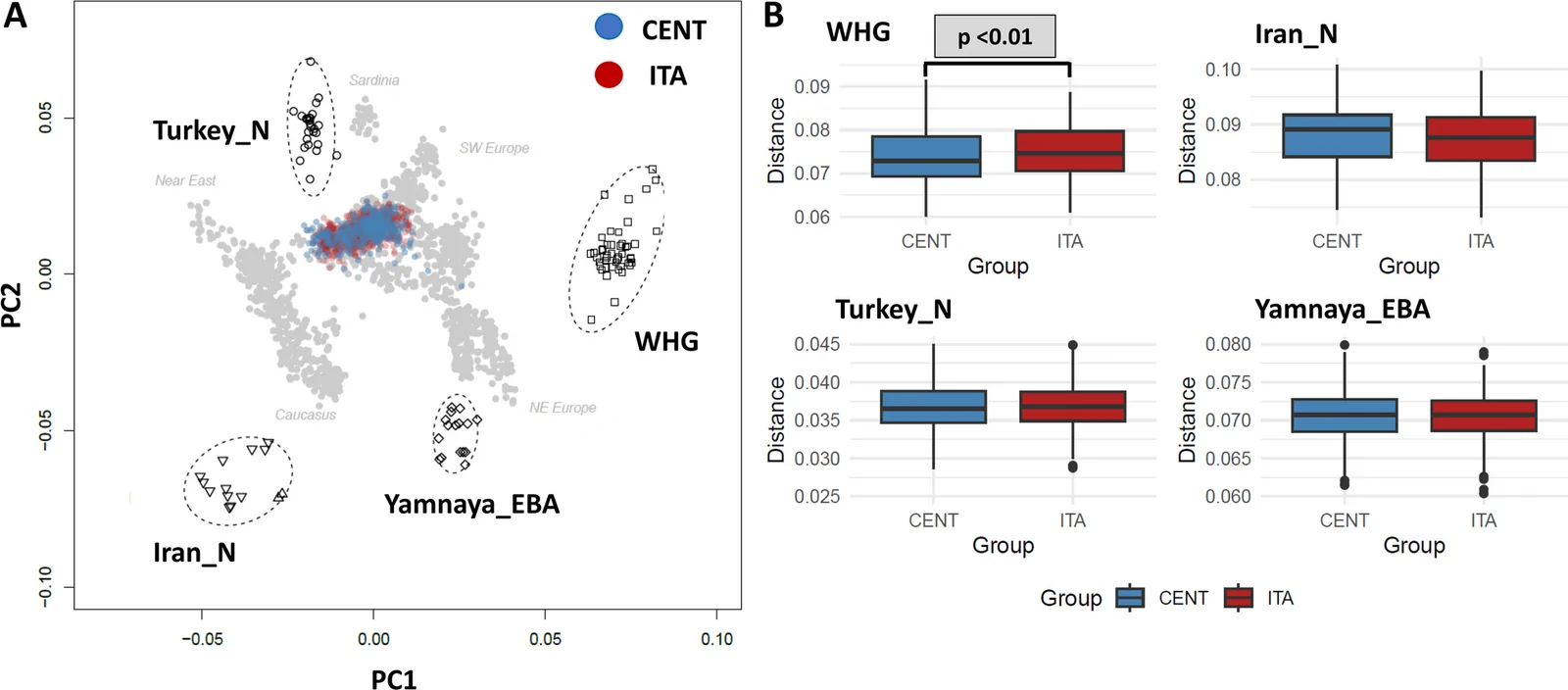
Results of the PCA projection analysis. **A** Scatterplot with genetic components of 333 *CENT*, 690 *ITA*, and selected ancient individuals from the four basal ancient ancestral groups (47 *WHG*, 14 *Iran_N/CHG*, 26 *Turkey*_*N*, and 16 *Yamnaya*_*EBA*) projected onto the modern Euro-Mediterranean reference space. **B** Boxplots displaying the distribution of Euclidean distances between *CENT* or *ITA* individuals and the centroid of each of the four considered ancestral groups

A Principal Component Analysis (PCA) was initially performed on the merged genotyping dataset including the Italian *CENT* and *ITA* as well as the selected ancient individuals, by projecting the genetic components onto the space obtained from a panel of modern reference western Eurasian populations (Supplementary File 1: Supplementary Table 2, Supplementary File 2: Supplementary Fig. 1) extracted from the AADR Human Origins database [27]. Overall, the obtained PCA plot confirms the geographic and genetic matching between *CENT* and *ITA* groups (Fig. 2A), reflecting the North–South latitudinal cline of genetic variation previously described for the Italian Peninsula and its positioning within the Euro-Mediterranean genetic landscape [21–23].

Previous genomic investigations suggested the genetic composition of present-day Italians to have been shaped by differential ancient genetic components. Above the contribution of hunter-gatherer-related ancestry, a significant impact of the expansion of Anatolian Neolithic farmers was detected in all the Peninsula. At the same time, two differential contributions during the Bronze Age were shown to have continued to shape the gene pool of the groups distributed along Italy in post-Neolithic times [21, 22]. One of these processes was the well-characterized Yamnaya-related signature associated with the expansion of nomadic groups from the Pontic-Caspian Steppes throughout Continental Europe, which is higher in northern Italy. The second contribution was instead associated with an ancestry related to Iranian Neolithic and Caucasian hunter-gatherers, possibly carried along the Mediterranean at the same time but independently from the Yamnaya-Steppe migrations, that is predominantly observed in Southern Italy [21–23, 26].

Potential differences between *CENT* and *ITA* in their relationships with respect to these ancestral groups that were demonstrated to have formed the current genetic make-up of Italians were preliminarily evaluated by measuring Euclidean distances between centenarian or control individuals and the centroids of the four ancestral populations within the obtained bidimensional space defined by the first two principal components (PC1 and PC2) (Fig. 2B). While no significant differences were observed between *CENT* and *ITA* distances to the *Turkey_N*, *Iran_N*, or *Yamnaya_EBA* centroids, statistical comparisons using Wilcoxon tests revealed that *CENT* individuals appear significantly closer to the *WHG* centroid than *ITA* controls (*p*-value = 9.44e-03). The same result was replicated in the sex-specific analysis when repeated on the female subset (*p*-value = 5.90e-03), confirming the pattern observed in the combined dataset.

This observation was also substantiated by *f*-statistics tests in the form of *D (CENT, ITA; Ancient, Mbuti).* Accordingly, comparisons between the 333 *CENT* and a subset of a comparable number of 345 *ITA* controls, randomly subsampled across 1,000 iterations while maintaining the overall genetic, geographic, and sex distribution within the subsampled group, showed the only significant result being observed for the *WHG* ancestry, with an expected Z-value of 6.08, thus indicating that *CENT* are more genetically similar to *WHG* than *ITA*. No significant Z-values were instead observed for the other three ancestral populations, namely *Turkey_N*, *Iran_N*, and *Yamnaya_EBA* (Supplementary File 2: Supplementary Fig. 2). Consistent with PCA findings, the *f*-statistics calculated using female individuals yielded significant values (Z > 3) exclusively for the *WHG* component, thus supporting also in sex-specific analyses a stronger *WHG* affinity among female centenarians.

### Association between ancestry proportions and centenarian status

To further explore the contribution of *WHG* ancestry, a supervised admixture analysis was subsequently performed, using the four ancient ancestral groups as parental components to directly estimate and compare the ancestry proportions of the 333 centenarians and 690 controls, resulting in the following mean values for *CENT* (*WH*G = 0.022, *Iran_N* = 0.019, *Turkey_N* = 0.63, *Yamnaya_EBA* = 0.32) and *ITA* (*WHG* = 0.017, *Iran_N* = 0.022, *Turkey_N* = 0.63, and *Yamnaya_EBA* = 0.33), respectively (Supplementary File 2: Supplementary Fig. 3). Logistic regression models adjusted for PC1, which captures the north–south pattern of distribution of genetic variation within the Italian population, were then applied to investigate possible associations between the estimated proportions of ancestral components and the centenarian status, after having standardized all continuous variables (see Materials and Methods for details). The obtained results revealed a significant effect only for the *WHG* ancestry (Table 1, Supplementary File 1: Supplementary Table 4). In particular, the estimated coefficient for *WHG* was 0.32 (95% CI: 0.07; 0.57, OR = 1.38, *p*-value = 1.23e-02), thus indicating that a 1-standard deviation (1-SD) increase in the *WHG* component is associated with a 38% increase in the odds of being a centenarian. When the analysis was repeated on the female subset, the *WHG* coefficient remained significant and was even stronger (*β* = 0.87, 95% CI: 0.53; 1.22, OR = 2.38, *p*-value = 6.31e-07), thus confirming the association observed in the full dataset. Notably, in the female-specific analysis, the *Yamnaya_EBA* component also reached statistical significance (*β* = –0.37, 95% CI: –0.60; –0.14, OR = 0.69, *p*-value = 1.67e-03) with an opposite effect to that of *WHG*, suggesting a negative association between Yamnaya ancestry and the probability of being a centenarian in the female group.

**Table 1:** Results of the logistic regression models for the four ancestral components (AC)

AC	Estimate (ß)	Std. error	z-value	*p*-value	95% CI lower	95% CI upper	OR	Significance
**WHG** (*N* = 47)	0.32	0.13	2.50	1.23e-02	0.07	0.57	1.38	*
**Iran_N** (* N* = 14)	−0.03	0.17	−0.21	8.29e-01	−0.37	0.30	0.97	
**Turkey_N** (* N* = 26)	0.01	0.09	0.06	9.54e-01	−0.17	0.18	1.01	
**Yamnaya_EBA** (* N* = 16)	−0.18	0.11	−1.56	1.18e-01	−0.40	0.04	0.68	

Significance codes: *p < 0.05

To complement the logistic regression findings, a residual-based predictive analysis was also performed. In fact, while logistic regression models directly assess the association between ancestry proportions and centenarian status, the residual-based approach was instead aimed at identifying differences in ancestry proportions that are independent from the population genetic structure of Italy. To address this, we first tested the correlations between ancestry components and population genetic structure (represented by the PC1 coordinates) in a randomly selected subset of 345 *ITA* individuals, which served as the training dataset. We ensured that genetic, geographical, and sex distributions in this training set were matched with those of the remaining 345 *ITA* controls, which were used as the testing dataset (see Materials and Methods section and Fig. 1). Positive correlations were identified between PC1 with *WHG* (*r*^2^ = 0.87) and *Yamnaya_EBA* (*r*^2^ = 0.78), whereas negative correlations were observed between PC1 with *Iran_N* (*r*^2^ = −0.91) and *Turkey_N* (*r*^2^ = −0.70), thus indicating higher proportions of *WHG* and *Yamnaya_EBA* ancestry at higher PC1 values, corresponding to *ITA* individuals from Northern and Central Italy, while opposite South–North trends can be observed for the *Turkey_N* and *Iran_N* ancestry proportions, in agreement with previous observations on the ancient genetic legacy of the Italian population [21–23, 40].

**Fig. 3:**
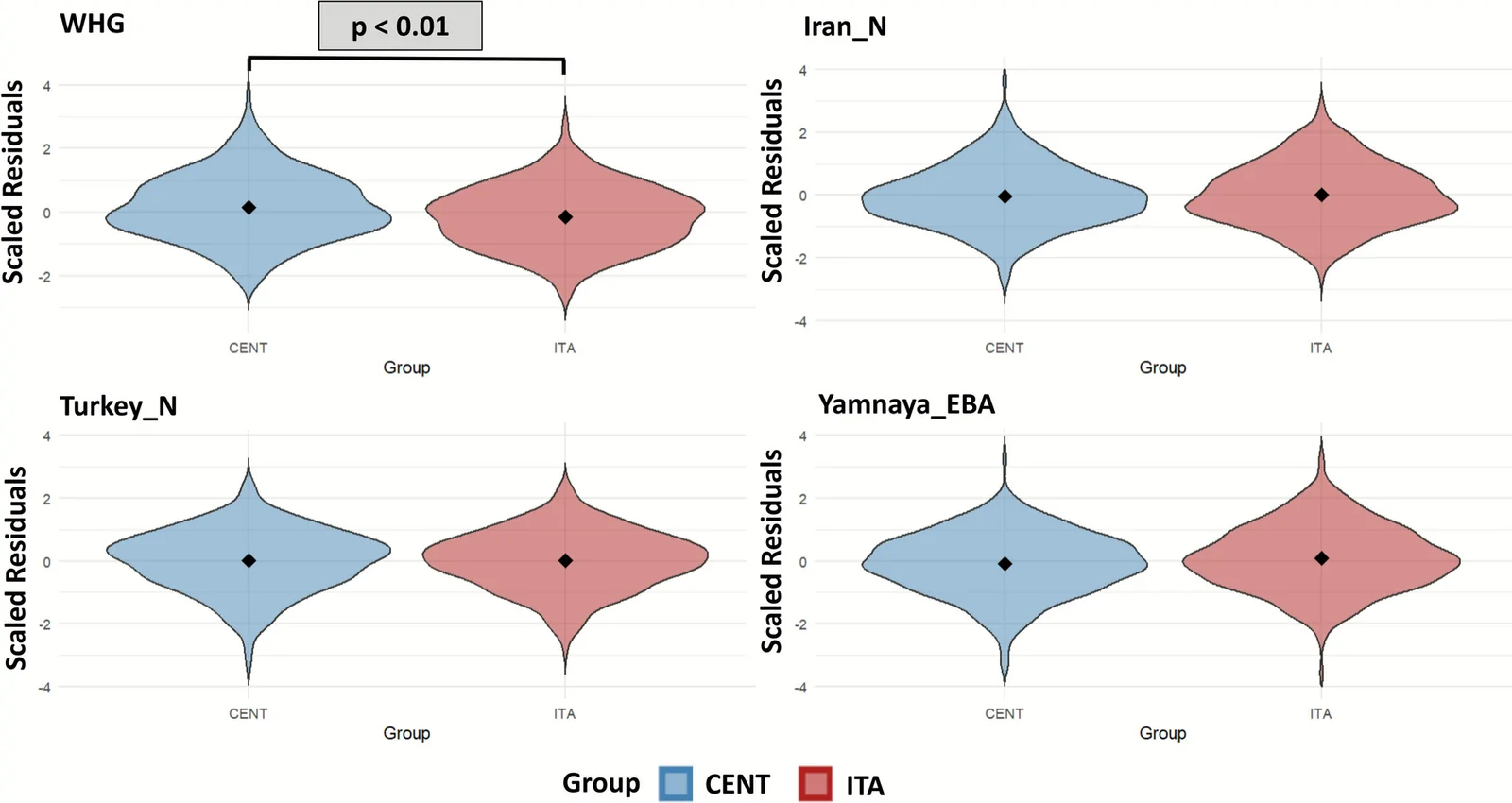
Violin plots displaying the distributions of scaled residuals for each ancestral population. Differences between the two analyzed modern Italian group (*CENT* and *ITA*) were evaluated using the *t*-test (*WHG* and *Yamnaya_EBA*) and Wilcoxon non-parametric test (*Iran_N* and *Turkey_N*), after having assessed for the normality assumption of the distributions

Linear regression models were then fitted in the training dataset to predict each ancestry component based on PC1 (i.e., the structuring pattern of genetic variation along the Italian Peninsula), and the obtained models were applied to the testing *ITA* and *CENT* datasets for computing residuals, representing deviations from the predicted ancestry proportions. Overall, *CENT* individuals exhibited significantly higher mean residuals for *WHG* compared to the baseline expectation derived from the training dataset (mean residual significantly greater than zero with a *p*-value < 0.01). In contrast, the testing *ITA* dataset showed no significant deviation from the baseline (mean residual not significantly different from zero). For the other ancestral components (*Iran_N*, *Turkey_N*, *Yamnaya_EBA*), residuals in both *CENT* and testing *ITA* datasets were not significantly different from zero, thus indicating no substantial deviations from the baseline expectation. These results suggest that centenarians exhibit a distinct *WHG* ancestry signal that cannot be fully explained by the population genetic structure captured by PC1. Furthermore, direct comparison of residuals distributions between *CENT* and *ITA* revealed significantly higher mean values in *CENT* individuals compared to the testing *ITA* controls (*p*-value < 0.01) for the *WHG* ancestry, while this enrichment was not observed for any of the other ancestry components (Fig. 3).

### Analysis of ancestral composition and pro-longevity SNPs

**Fig. 4:**
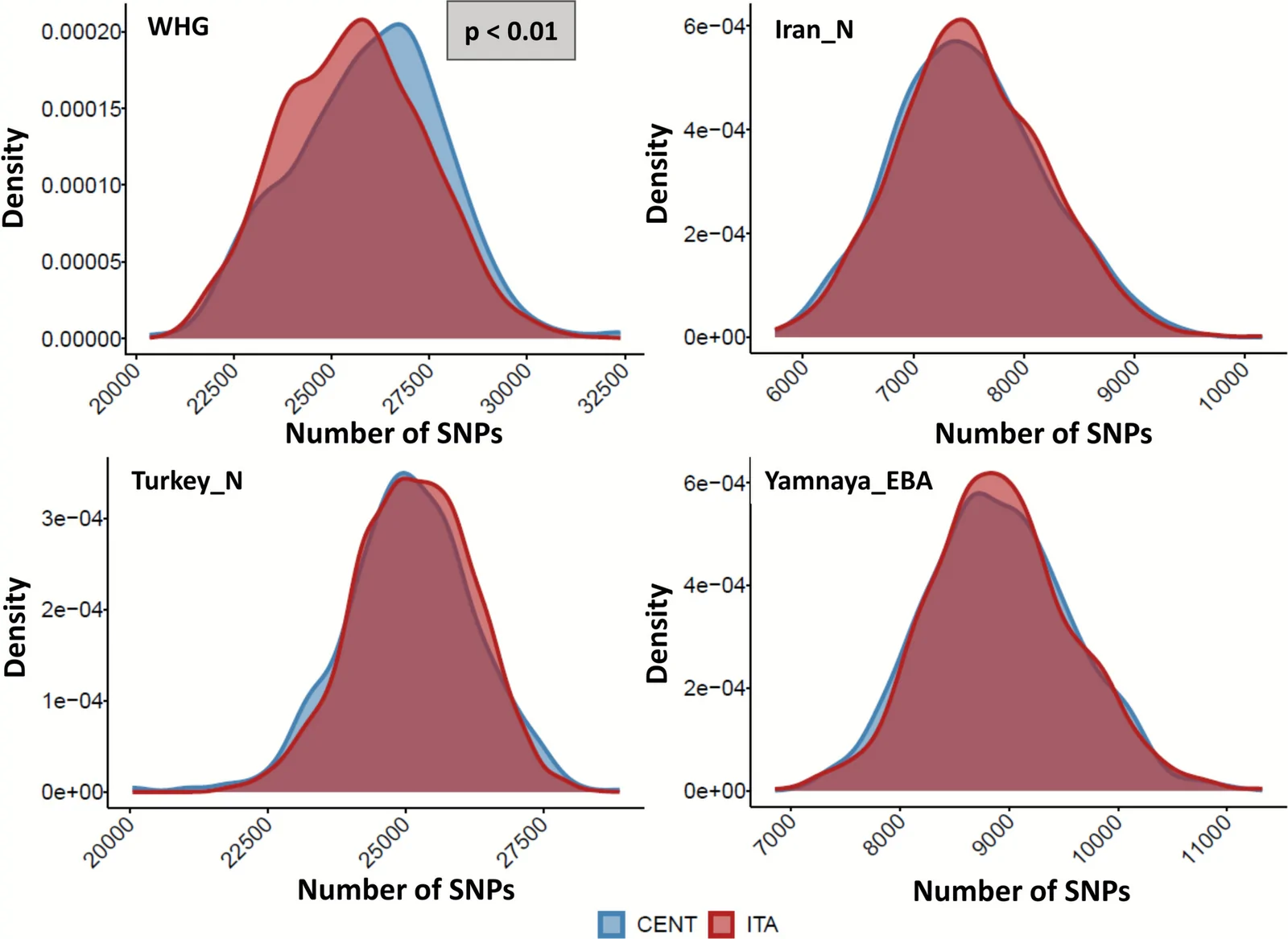
Density plots displaying the distributions of the total number of SNPs assigned to each ancestral population (*WHG*, *Turkey_N*, *Yamnaya_EBA*, and *Iran_N*) as determined by ChromoPainter copying probabilities. The computed density on the y-axis represents the probability density of individuals exhibiting a given number of SNPs attributed to a specific ancestry. A SNP was assigned to an ancestry when the probability of copying from the corresponding donor population exceeded 0.85. Differences between *CENT* and *ITA* were assessed using *t*-tests, and significant *p*-values are reported in the plots

To infer ancestry tracts in the genomes of present-day individuals inherited from a set of source populations, we conducted a ChromoPainter (CP) analysis on the combined dataset consisting of modern and ancient individuals, by considering the 333 centenarians (*CENT*) and 690 Italian controls (*ITA*) as two distinct recipient populations, and the four main ancient ancestral groups, i.e., *WHG*, *Turkey*_*N*, *Yamnaya_EBA*, and *Iran_N*—as donor populations. In particular, for each recipient individual, we assigned an ancient-specific ancestry to each SNP with an expected probability of copying from the respective donor population greater than 0.85, and then we examined the number of SNPs identified as having *WHG*, *Turkey_N*, *Yamnaya_EBA*, or *Iran_N* ancestry. Overall, the *t*-tests revealed that the number of SNPs associated with *WHG* ancestry was significantly higher in *CENT* individuals compared to *ITA* controls (*p*-value = 5.02e-05), whereas no significant differences were observed for the other ancient ancestries (Fig. 4). In particular, the mean number of *WHG* SNPs in *CENT* was 25.945,99 with a standard deviation of 1.921,69, while in *ITA*, the mean number was 25.582,18 with a standard deviation of 1.836,24. Also in this case, when sex-specific analysis was repeated on female individuals, centenarian women similarly showed a significantly higher number of *WHG-*associated SNPs than female controls (*p*-value = 3.12e-04).

**Fig. 5:**
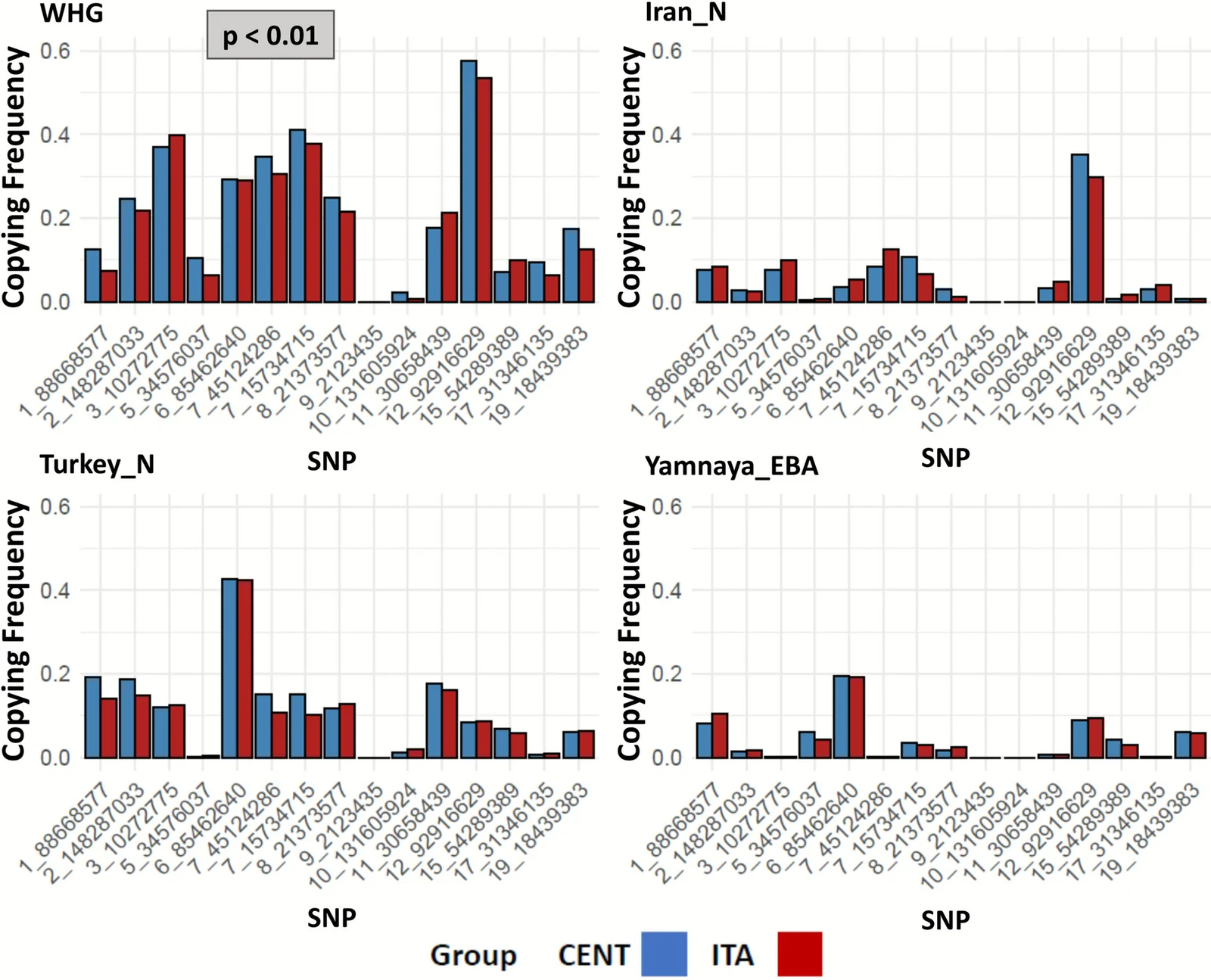
Frequency of the cumulative number of alleles inherited from each ancestral population at the 15 pro-longevity SNPs for the *CENT* and *ITA* groups

Finally, we specifically focused on 18 pro-longevity SNPs (Supplementary File 1: Supplementary Table 3) that were significantly associated with longevity (*p*-value < 1.00e-04 and OR > 1) from the association analysis between *CENT* and *ITA* (Giuliani et al., 2018). Before performing the burden test, we examined the LD structure among variants belonging to the same haploblock and identified three pairs of SNPs in high linkage disequilibrium: 1_88668577 and 1_88710048 (*R*^2^ = 0.70), 6_85462640 and 6_85417046 (*R*^2^ = 0.88), and 7_45124286 and 7_45122723 (*R*^2^ = 1). To prevent inflation of the burden signal, one SNP from each pair was excluded, resulting in 15 independent pro-longevity SNPs retained for the analysis. Burden tests were then performed to assess whether the cumulative number of alleles inherited from each ancestry at the 15 independent pro-longevity SNPs differed between *CENT* and *ITA*. Significant results were observed only for the *WHG* ancestry (Fig. 5), with the total number of *WHG* inherited alleles at the pro-longevity SNPs being significantly higher in *CENT* than in *ITA* (*p*-value = 9.96e-04), while no significant differences were observed for the other remaining ancestries (*Turkey_N*, *Iran_N*, *Yamnaya_EBA*). Notably, when the analysis was repeated on the female subset, the *WHG* ancestry remained significant (*p*-value = 1.21e-05).

## Discussion

The genetics of human longevity has been largely explored using classical methods primarily developed in the field of genome-wide association studies [4]. While these studies have contributed to identifying and describing the genetic determinants of human longevity [3–6], they have provided limited insights into the evolutionary and genomic history of the genetic background favoring this trait.

Recent studies, driven by the advancements in the field of paleogenomics and aDNA analysis, have attempted to investigate and reconstruct the role of ancient ancestries in shaping present-day phenotypes [8, 11, 15]. However, none of these studies have specifically focused on longevity. In fact, most of the biobanks used in these investigations, despite being among the largest available to date, often rely on cohorts that do not represent long-lived individuals and centenarians, thereby limiting the ability to reconstruct the contribution of these ancient ancestral components to the rigorous longevity phenotype.

In the present study, we propose and employ an evolutionary approach based on population genetics and paleogenomics to further investigate this phenomenon and describe how ancient ancestral genetic components may have contributed to the present-day longevity landscape.

We based our study on a rigorous definition of longevity [3, 16], specifically including individuals who survive beyond the age corresponding to the 99th survival percentile of their birth cohort. Recent studies have indeed emphasized the importance of defining the percentile of survival in all studies on longevity, because it has been shown that even small differences in survival percentiles are likely to have profound effects on the genetic contribution to this trait [16]. The genetic basis of surviving to the 5th percentile is likely to be weaker and different from the genetic basis of surviving to the 1 st percentile, which in turn is different from those for rarer percentiles of survival. By focusing on this cohort, we thus aimed to maximize the role of genetic determinants, while minimizing the effects of non-genetic/environmental factors.

Furthermore, to reduce the heterogeneity caused by varying population genomic histories, we focused on the Italian Peninsula as representative of the Southern European genetic cluster. It is well known that Continental and Southern Europe have experienced distinct migration patterns since prehistoric times [23, 41, 42]. At the same time, the Italian Peninsula, thanks to its unique geographic position, can serve as an ideal case study because Italian genomes capture several ancient genetic signatures resulting from past migration processes and admixture events [21–23]. The current genetic background of modern Southern Europeans (and of the Italian population) has been indeed shown to include a combination of at least three main ancient genetic components, derived mainly from (i) Mesolithic Western European Hunter-Gatherers (WHG), (ii) early Neolithic Farmers from the Near East, and (iii) Early Bronze Age herders from the Pontic-Caspian Steppes, with present-day Italians capturing also a possible non–Steppe contribution derived from Caucasian hunter-gatherers and Iranian Neolithic-related ancestry [22, 23, 26].

Our analyses, which compared 333 Italian centenarians and 690 healthy controls matched for sex and micro-geographic origin [7] to 103 ancient genomes representative of the main population ancestries that contributed to the present-day European genetic make-up over the last 20,000 years, showed for the first time that long-lived individuals exhibit a higher affinity to Western European Hunter-Gatherer (*WHG*)-related ancestry. This result is supported by various analytical approaches that accounted for the population structure of Italy, thus suggesting a contribution of *WHG* ancestry independent of the geographic structuring of the Italian genetic variation. Accordingly, logistic regression models revealed a significant positive association between *WHG* ancestry and the centenarian status, even after adjusting for population structure, whereas no significant results were observed for the other ancestries. Complementing these findings, the residual-based predictive analysis, which focused on the deviations in ancestry proportions that could not be explained by the underlying population genetic structure, consistently showed that centenarians exhibited significantly higher *WHG* ancestry proportions compared to the baseline expectation. We note that this analysis was conducted while accounting for population structure, in line with our hypothesis aimed at isolating the role of ancestral components. However, we do not exclude the possibility that aspects of genetic structure may also be informative for the genetic determinants of longevity, and future studies will be needed to explore this direction further.

By painting the chromosomes of modern Italian samples using the genomes of the ancient ancestral groups as donors, we also showed that Italian centenarians reveal a significantly higher number of SNPs of *WHG*-related ancestry compared to controls. Furthermore, when we focused on pro-longevity SNPs and performed burden tests, we observed significant results only for the *WHG* ancestry, with the cumulative number of *WHG* inherited alleles at the pro-longevity SNPs being significantly higher in *CENT* than in *ITA*.

Western European Hunter-Gatherers (WHG) are recognized as an ancestral component contributing to the ancestry of present-day Europeans, descending from Mesolithic hunter-gatherer populations occupying southern and central-western Europe. This group has been defined based on genetic affinity among individuals who largely replaced pre-existing European populations after the Last Glacial Maximum. In particular, this ancestry is associated with the ∼14,000-year-old individual from Villabruna, Northern Italy [43]. The appearance of the Villabruna-Cluster/WHG ancestry has been linked to population shifts within Europe at the end of the Ice Age. In particular, the expansion of this ancestry has been correlated with the period of rapid climate warming of the Bølling-Allerød interstadial period and culturally with the Epigravettian transition in southern Europe and the Magdalenian-to-Azilian transition in western Europe.

These data support our findings published in 2018 [7], which were based on biodemographic analyses using only modern human populations. When projected onto the genetic variation of Italians, the centenarians did not exhibit a strong structure and appeared instead to overlap with Central/Southern Italians. Since the Italian genetic structure was predominantly shaped by Neolithic and post-Neolithic dynamics that contributed differentially to the gene pool of the groups distributed along the peninsula, in our previous paper we had hypothesized that some pre-Neolithic components might be favorable for longevity today. In this paper, with the availability of ancient DNA data, we can more precisely suggest these “pre-Neolithic components” as the WHG (Western Hunter-Gatherer)–related ancestry.

Thus, our findings can be interpreted as reflecting, on one hand, ancient WHG-related variants that may currently favor longevity, and on the other, they align with evidence showing that genetic changes occurred since the Neolithic may have introduced alleles that, while beneficial in past environments, may now be associated with increased susceptibility to modern diseases. A particularly relevant example involves the inflammatory component. The rise in pro-inflammatory alleles after the Neolithic likely reflects adaptive responses to increased pathogen exposure, population density, and changes in diet and lifestyle [10, 44]. However, while these variants promoted survival and reproduction in past infectious environments, they may now contribute to inflammaging and age-related chronic diseases in contemporary industrialized populations [45]. Although no single post-Neolithic ancestry component showed a strong negative association with longevity, the possibility of small cumulative influences cannot be excluded. The fact that, in the female-specific association analysis, the Yamnaya ancestry is negatively associated with longevity may illustrate this possibility. Further data will be essential to clarify these complex evolutionary dynamics and to investigate the role of specific biological pathways and their evolutionary history, which could further illuminate mechanisms contributing to human longevity.

Our results may appear to contradict those described in other studies [11, 12], which found a higher frequency of APOE4 in WHG populations. However, the history of epsilon variants of APOE is very unique due to the complexity of this gene in terms of its evolutionary history. Therefore, when interpreting these findings, it is important to note that the frequency of APOE4 in Southern European populations is lower than in Northern and Continental Europe, probably since ancient times. Specifically, Northern Europe has a relatively high frequency of APOE4 (15–21%), Central Europe shows intermediate frequencies (11–13%), whereas Southern Europe has the lowest frequencies (3–6%) [13]. In this context, representative WHG samples from different geographical areas are needed to draw robust conclusions and fully address this issue. To the best of our knowledge, there is currently a limited number of WHG individuals directly genotyped for both rs7412 and rs429358 (which define the APOE epsilon allele), with a different distribution between Northern and Southern Europe, which complicates the interpretation. It should also be noted that our study focuses on a Southern European population, where APOE4 is not among the strongest signals identified in GWAS and WGS studies of human longevity, often yielding marginal *p*-values [3, 7, 46, 47]. In this respect, recent studies have emphasized how distinct population-specific evolutionary and demographic histories contribute to influencing variability in the genetic determinants of longevity, thus highlighting the need for an approach that carefully considers population variation [1, 2].

In conclusion, this approach grounded in an evolutionary framework provides a historical-genomic perspective that reframes the concept of healthy ageing and longevity—not as a static or universal state, but as a dynamic phenotype shaped by the interplay of genomic population history and continually changing environmental contexts. The present study shows for the first time that the Villabruna cluster/WHG lineage, which has been linked to population shifts within Europe after the Last Glacial Maximum, contributes to longevity in the Italian population. We propose that the variants involved in this trait may have been introduced into the Italian gene pool at a very ancient time. In general, we have proposed here a new population-specific approach to the analysis of the genetic determinants of human longevity, exploiting the vast amount of data produced in the field of paleogenomics and taking into account biodemographic events that have occurred in the past. In this light, biodemographic history and genetic ancestry are not merely confounding factors in GWAS or in precision medicine studies, but important contributors to contemporary phenotypic variability.

## Supplementary Information

Below is the link to the electronic supplementary material.

ESM 1 (XLSX 426 KB)

ESM 2 (DOCX 426 KB)

## Data Availability

Genotyping data about Italian centenarians and their geographically matched controls is not publicly available due to privacy or ethical restrictions, and requests for access to raw data should be made to the corresponding author. BAM files for the comparison ancient genomes used in the present study were obtained at the European Nucleotide Archive (https://www.ebi.ac.uk/ena/browser/home) from publicly available sources. All codes used in the present study are available as functions that are implemented in publicly available software and R packages.
