# Supplementary material for: Western Hunter-Gatherer genetic ancestry contributes to human longevity in the Italian population
Source: GeroScience. 2025 Dec 13;48(5):7157–73. doi: 10.1007/s11357-025-02043-4 (PMC13601382; doi:10.1007/s11357-025-02043-4)
Supplement: Supplementary file 1 [file 11357_2025_2043_MOESM2_ESM.docx]

Supplementary file 2: Supplementary Figures

**Western Hunter-Gatherer genetic ancestry contributes to human longevity in the Italian population**

Stefania Sarno*, Vincenzo Iannuzzi*, Marco Sazzini, Chiara Pirazzini, Maria Giulia Bacalini, Davide Gentilini, Giuseppe Passarino, Daniela Mari, Daniela Monti, Benedetta Nacmias, Sandro Sorbi, Davide Pettener, Donata Luiselli, Claudio Franceschi, Paolo Garagnani, Cristina Giuliani

**Supplementary Fig. 1.** Principal component analysis on the modern Euro-Mediterranean samples extracted from the AADR HO panel that have been used as reference space in the projection analysis. Median coordinates and ± 2 standard deviations are represented for each given population.

**Supplementary Fig. 2.** Density probability distributions of Z-values for the four ancestral populations derived from f4-statistics tests across the performed 1,000 iterations. The expected Z-values for each ancestral population are indicated by the vertical dashed lines.

**Supplementary Fig. 3.** Ancestral component proportions estimated with the supervised ADMXITURE analysis for *CENT* and *ITA* using *WHG*, *Turkey_N*, *Iran_N*, and *Yamnaya_EBA* as parental components.
